# Cartilage-targeting engineered probiotic bacterial extracellular vesicles treat osteoarthritis through modulation of matrix homeostasis

**DOI:** 10.1016/j.mtbio.2026.103668

**Published:** 2026-09-24

**Authors:** Luyu Niu, Wanzhuo Chen, Tong Liu, Xiaoyi Liang, Peiran Song, Han Liu, Teng Su, Xiao Chen, Zhongtang Liu, Yingying Jing, Jin Cui, Jiacan Su

**Affiliations:** a Department of Orthopedics, Changhai Hospital, Naval Medical University, Shanghai, China; b Department of Orthopedics, Xinhua Hospital, Shanghai Jiao Tong University School of Medicine, Shanghai, China; c Institute of Translational Medicine, Shanghai University, Shanghai, China; d MedEng-X Insititutes, Shanghai University, Shanghai, China; e National Center for Translational Medicine (Shanghai) SHU Branch, Shanghai University, Shanghai, China

**Keywords:** Osteoarthritis, Bacterial extracellular vesicle, Intra-articular drug delivery, Targeted therapy, Extracellular matrix

## Abstract

Reversing extracellular matrix (ECM) metabolic dysregulation to facilitate cartilage repair represents a key therapeutic strategy for osteoarthritis (OA) treatment. Owing to the avascular nature of joint structures, intra-articular injection is a commonly used clinical administration route. However, the current drug retention time is insufficient to effectively reverse OA progression. In this study, we demonstrated that natural bacterial extracellular vesicles (BEVs) are capable of repairing cartilage damage. We modified *Lactobacillus rhamnosus* GG (LGG)-derived BEVs with a chondrocyte-targeting peptide to prolong their therapeutic efficacy. Engineered BEVs, as a drug delivery system, are capable of continuously transporting endogenous miRNAs. Cartilage-targeting LGG-EVs (W-LGG-EVs) penetrate deeply into cartilage tissue and promote extracellular matrix secretion by chondrocytes. Furthermore, W-LGG-EVs were shown to restore cartilage homeostasis and ultimately alleviate stress-related OA. BEVs, as a novel and safe therapeutic agent, hold clinical feasibility.

## Graphical abstract

**Figure:**
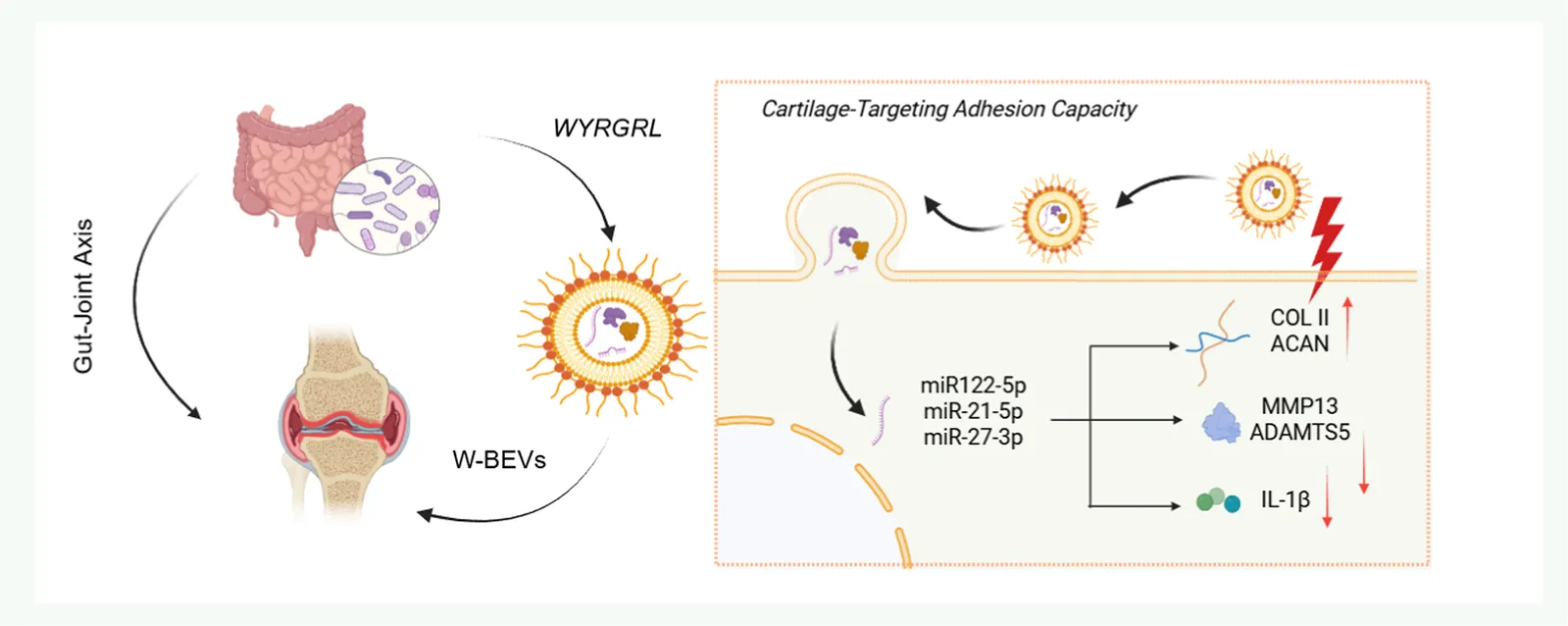

## 1 Introduction

Osteoarthritis (OA) is a common degenerative musculoskeletal disorder among middle-aged and elderly individuals. It leads to joint swelling, stiffness, and limited mobility, resulting in disability in patients [1]. OA affects the entire joint, leading to subchondral bone remodeling, osteophyte formation, and inflammation [2,3]. Unfortunately, current treatments for OA still lack the ability to achieve long-term, satisfactory, and effective intervention. Currently, clinical drugs for OA can only subjectively alleviate symptoms without fundamentally hindering the progression of the disease [4]. Joint replacement surgery places great physical and economic strain on patients and is frequently considered the last treatment option. There is an urgent clinical need for the development of tissue regeneration methods to prevent joint degeneration, repair damaged cartilage, and restore joint function.

In recent years, novel cell-free therapies based on extracellular vesicles (EVs) have been gradually applied to the treatment of degenerative diseases. EVs are nano-sized membrane-enclosed vesicles, with diameters between 30 and 150 nm. EVs are actively released from cells under either physiological or pathological circumstances. As endogenous mediators of intercellular communication, EVs can deliver bioactive components including proteins, lipids, and microRNAs, thus modulating the functions of recipient cells [5]. Studies have found that EVs derived from various stem cells, such as mesenchymal stem cells and umbilical cord mesenchymal stem cells, can alleviate OA progression through anti-inflammatory, immunomodulatory, and other mechanisms [[6], [7], [8]]. Additionally, in 2025, clinical-grade EVs originating from umbilical cord mesenchymal stem cells were first clinically validated in human joints, indicating that EVs have become a promising therapeutic candidate for OA [9]. However, in actual clinical testing, the clinical translation of mammalian cell-derived EVs has been found to face numerous challenges, including long production cycles, low yields, high costs, substantial batch to batch variability, and potential immunogenicity issues.

In contrast to mammalian cell-derived EVs, BEVs enable large-scale, low-cost, and standardized production via fermentation engineering, thereby demonstrating substantial potential for clinical translation [[10], [11], [12]]. In addition, accumulating evidence has demonstrated that probiotic-derived bacterial extracellular vesicles (BEVs) possess therapeutic potential against conditions including atherosclerosis and Alzheimer's disease [13,14]. Meanwhile, as the human body is a complex ecosystem, the gut-joint axis linking the resident microbiota with joint health has also been confirmed [15]. BEVs serve as critical mediators in the crosstalk between the gut microbiota and the host, thus occupying a pivotal position within this axis. Studies have found that EVs released by *Lactobacillus johnsonii* are capable of restoring the metabolic function of synovial and cartilage tissues via immunomodulation and suppression of inflammation [16]. Therefore, utilizing natural BEVs derived from probiotics to treat osteoarthritis may represent a promising strategy. The avascular nature of articular cartilage leads to poor biodistribution of systemically administered drugs [17]. Intra-articular injection is a rational method for enhancing joint bioavailability. Nevertheless, within hours of intra-articular administration, the majority of the drug is cleared via the synovial fluid into the blood or lymphatics [18]. The remaining drug is further sequestered from chondrocytes by the dense cartilage matrix [19]. The combination of synovial fluid turnover and the compact matrix network imposes a substantial spatial obstacle, limiting the ability of delivered drugs to sustain therapeutic concentrations over time [20]. Consequently, ensuring prolonged BEVs retention with the joint cavity is critical. Surface modification of BEVs enables targeted drug delivery [21,22]. Engineering BEVs provides the possibility of developing ideal therapeutics for OA treatment.

Previously, we successfully developed bone-targeting BEVs for osteoporosis treatment [23]. To further investigate the therapeutic potential of probiotic BEVs in OA, we offer a novel and innovative approach for OA based on the gut–joint axis (Fig. 1). We engineered cartilage-targeting *Lactobacillus rhamnosus* GG derived extracellular vesicles (LGG-EVs) with cartilage homeostatic modulation capabilities to attenuate OA progression in an anterior cruciate ligament transection (ACLT) murine model. LGG-EVs contain a large number of miRNAs associated with chondrocyte metabolism. Meanwhile, we developed a strategy to conjugate BEVs with the chondrocyte-targeting peptide WYRGRL. W-BEVs can efficiently target chondrocytes for drug delivery, enhancing vesicle retention within the cartilage layer. Meanwhile, BEVs can reverse progressive cartilage degeneration, thereby controlling stress-induced OA. In conclusion, this engineered W-LGG-EVs offer a promising and effective clinical intervention for OA.

**Fig. 1:**
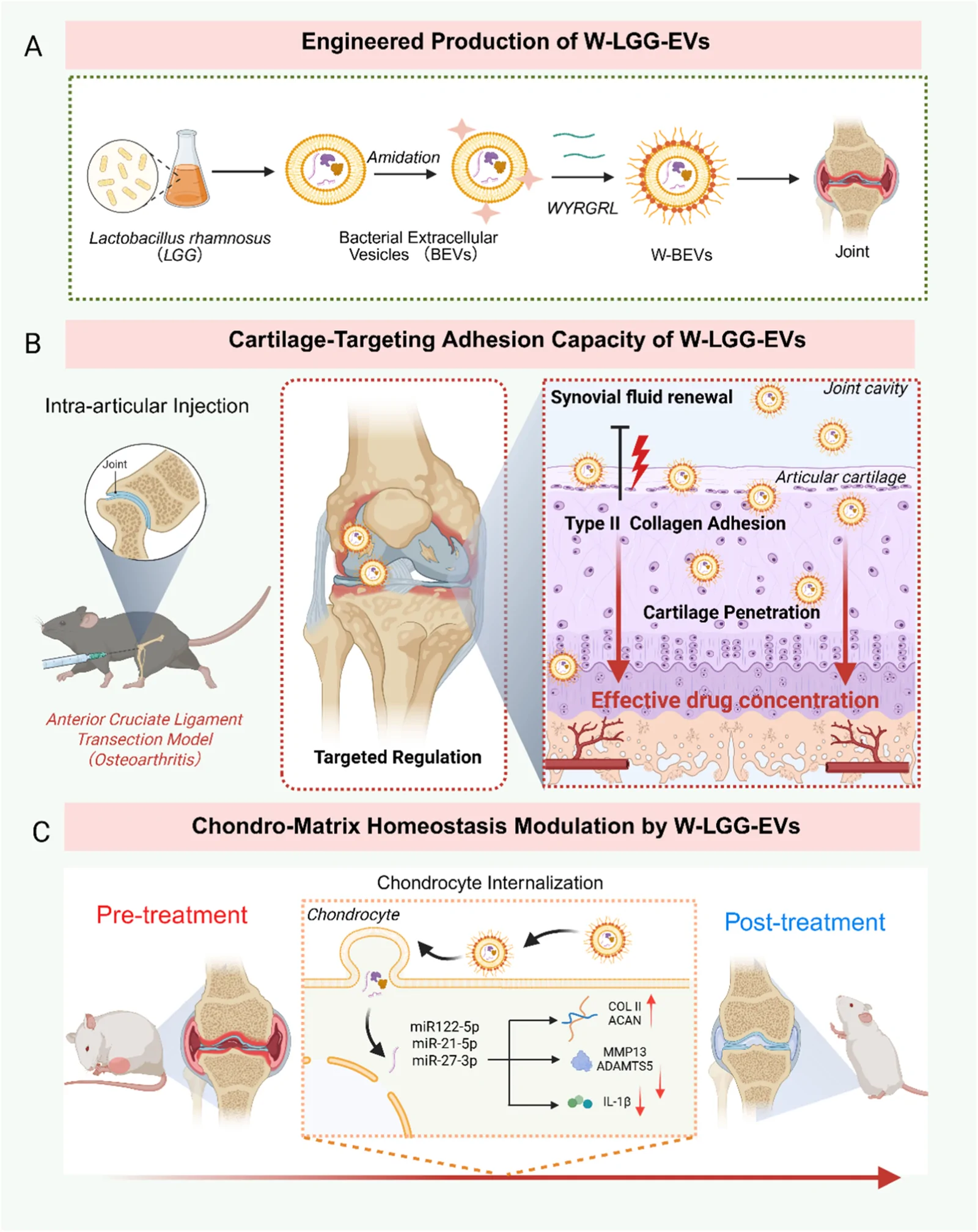
Preparation of W-LGG-EVs and proposed Anti-Osteoarthritis Mechanism. Supernatant from the probiotic LGG was collected, and LGG-EVs were isolated. The targeting peptide WYRGRL was subsequently anchored onto the membrane of LGG-EVs to generate cartilage-targeting W-LGG-EVs for OA treatment. W-LGG-EVs achieve prolonged retention and deep penetration within articular cartilage by binding to type II collagen. Subsequently, W-LGG-EVs deliver endogenous miRNAs to enhance extracellular matrix (ECM) secretion by chondrocytes, ultimately attenuating cartilage damage in OA.

## 2 Material and method

### 2.1 Bacterial culture and BEVs isolation

Frozen LGG stock (ATCC 53103) was obtained from Liu Han at the Shanghai University. LGG was grown anaerobically in De Man, Rogosa, and Sharpe (MRS) broth. LGG was cultured in 0.22-μm filter-sterilized MRS broth. The primary fermentation culture was obtained by incubating the cells at 37 °C for 24 h under static, aerobic conditions. This culture was then inoculated into 500 mL of fresh MRS broth at a 1:500 ratio and further incubated at 37 °C with shaking at 220 rpm for 48 h. The culture medium was collected for BEVs extraction.

To remove bacterial debris, the fermentation broth was subjected to high-speed centrifugation (10,000×g, 1 h). Collected culture was filtered via filters to remove large particulate impurities. The concentrated solution was then ultracentrifuged in an ultracentrifuge tube (100,000×g, 3 h) to pellet the crude BEVs. Resuspension of the pellet was carried out in PBS and further purified by another round of ultracentrifugation (150,000×g, 3 h), yielding highly purified LGG-EVs.

### 2.2 Modification of LGG-EVs with targeting peptide

LGG-EVs were resuspended in HEPES buffer (Gibco, USA). Then, 2 μL of methacrylic anhydride (Sigma, Germany) was introduced. The mixture was stirred for 24 h to yield EVs modified with alkenyl double bonds. A 0.22 μm syringe filter was used. And BEVs were harvested again via ultracentrifugation to remove the unreacted methacrylic anhydride. The modified EVs were redissolved in buffer, thoroughly mixed with 1 mg of the peptide WYRGRL. The process allowed the alkenyl double bonds on the EVs to undergo an acylation reaction with the amino groups on the peptide. After 24 h of incubation at 4 °C with stirring, the peptide conjugated to the EVs. Any unbound peptide was removed using an ultrafiltration concentrator, and final collection of the engineered BEVs (W-LGG-EVs) was achieved by ultracentrifugation. The targeting peptide was conjugated with FITC. Following binding to the vesicles, the mixture was subjected to ultracentrifugation to eliminate unbound fluorescent peptides, thereby preventing fluorescence interference from free peptides. The binding efficiency of the peptide was subsequently assessed in the resuspended pellet using nano-flow cytometry (N30E NanoFCM, China).

### 2.3 Characterization of LGG-EVs

The BEVs suspension was adsorbed for 15 min by copper grid. After fixation with paraformaldehyde, the sample was stained with 2% uranyl acetate. Vesicle images were captured by a transmission electron microscope (TEM) (HITACHI, Japan). The diluted BEVs were subjected to size distribution analysis using a nanoparticle tracking analysis (NTA) system (Particle Metrix, German). The surface charge (zeta potential) was assessed by a Zetasizer (MALVERN, UK).

### 2.4 Primary chondrocyte isolation

Full-thickness cartilage obtained from neonatal mice serves as a source of primary chondrocytes. The isolated cartilage was then immediately immersed in PBS to ensure tissue viability during subsequent processing. The minced cartilage was digested and centrifuged. The chondrocytes were then seeded in complete medium to obtain mature cells for subsequent experiments (Fig. S1A). Add 10 ng/mL IL-1β to the culture to induce an OA-like phenotype.

### 2.5 Cytotoxicity assessment

To evaluate in vitro cytotoxicity, both the CCK-8 assay (Beyotime, China) and the Live/Dead staining kit (Solarbio, China) were employed. Chondrocytes were plated in 96-well plates at 5000 cells per well. Following attachment, the cells were treated with different concentrations of LGG-EVs or W-LGG-EVs for 24 h. The cell viability was measured following the manufacturer's standard instructions.

### 2.6 Cartilage-targeting validation

The BEVs and W-BEVs were stained with DiR and administered into the joint cavity using microsyringe. The whole-body images were captured by in vivo imaging system (IVIS) (Biolight, China) at various time points under 750/780 nm conditions. At 3 days after injection, we harvested the knee joints and major organs from a subset of mice and examined them with the IVIS Spectrum system. Nuclear-stained knee joint sections were observed to evaluate the retention and accumulation of BEVs within the articular cartilage tissue.

To evaluate the cellular internalization of BEVs, LGG-EVs and W-LGG-EVs were fluorescently labeled using PKH-26. In brief, BEVs (1 × 10^10^ particles ml^−1^) were mixed with PKH-26 working solution and incubated in the dark for 30 min. The labeled BEVs were purified by filtration through a 0.22 μm pore-size membrane, followed by centrifuged at 100,000×g for 70 min to eliminate excess dye. The purified labeled vesicles were added to cultured cells and incubated for 24 h in the dark. After rinsing with PBS, cells were fixed with 4% paraformaldehyde for 10 min. The cytoskeleton was stained with Alexa Fluor 488–phalloidin (Sunride Biotech, China), and the nuclei were stained with DAPI. Finally, the samples were analyzed by fluorescence microscopy.

### 2.7 RT-qPCR analysis

After stimulating chondrocytes with IL-1β for 24 h, the cells were subsequently treated with either BEV or W-BEV. Cells harvested from the two treatment groups, along with those from the OA model and control groups, were collected for assessment of RNA expression levels. Total RNA was isolated from the washed adherent chondrocytes using TRIzol reagent according to the manufacturer's instructions. Briefly, one volume of TRIzol and 0.2 vol of chloroform were added to the cell lysate. After centrifugation, the aqueous phase was collected and mixed with an equal volume of isopropanol to precipitate the RNA. The RNA pellet was collected by centrifugation, washed with ethanol, and finally dissolved in DEPC-treated water for storage. The concentration was determined, and total RNA was reverse-transcribed into complementary DNA via a commercial reverse transcription kit (Vazyme, China). Quantitative PCR (qPCR) was then performed using the Maxima SYBR Green qPCR Master Mix (Thermo Fisher Scientific, USA) on a real-time PCR system, with GAPDH serving as the internal reference gene. The reaction setup and thermal cycling conditions followed the manufacturer's protocols. The relative gene expression levels were calculated using the 2^–ΔΔCt^ method. All primer sequences used are shown in Table 1.

### 2.8 Western blot analysis

Chondrocytes were lysed on ice with RIPA buffer for 30 min. The lysates were then centrifuged, and the supernatant was collected. Protein quantification was performed by the BCA Protein Assay Kit (Mei Duo Bio, China). Subsequently, the protein samples were heat-denatured for further analysis. The resolving and stacking gels were prepared according to standard protocols. Equal amounts of protein samples were then loaded into the wells and subjected to electrophoresis: initially concentrated at 80 V for 30 min, then resolved at 120 V for 1 h. After electrophoresis, the gel and the methanol-activated PVDF membrane were placed in close contact, and the proteins were transferred at a constant current of 260 mA for 2 h. The PVDF membrane was blocked with 5% skimmed milk for 2 h at room temperature. After blocking, the membrane was incubated overnight at 4 °C with primary antibodies directed against COL2 (MCE, USA, 1:1000), ACAN (Abcam, USA, 1:1000), ADAMTS5 (Abcam, USA, 1:2000), and MMP13 (Abcam, USA, 1:3000). The primary antibody was recovered after 12 h, and the membrane was washed before incubation with the appropriate secondary antibody. Finally, the membrane was washed again, and an appropriate amount of developing solution was added. The results were analyzed based on the exposure output from the imaging system.

### 2.9 Scratch wound healing assay and staining

The wound closure rate was defined as the ratio of the wound width at 24 h to the initial wound width at 0 h. Chondrocytes were uniformly seeded into plates and grouped. After the cells reached full confluence, we created a uniform straight scratch down the center of each well and washed away the detached cells, followed by co-incubation with LGG-EVs or W-LGG-EVs for 24 h. Bright-field images of the wound area were acquired at specified time points prior to and following the incubation period. The scratch width was measured at each time point using ImageJ software (NIH, USA). Safranin O (SO) and Toluidine Blue (TB) staining analyses followed published protocols. The staining kits (Solebao, China) were used to stain chondrocytes. After staining, the cells were imaged to assess proteoglycan content and chondrocyte morphology.

### 2.10 Animal osteoarthritis model

All animal procedures were performed in accordance with the guidelines approved by the Animal Ethics Committee of Shanghai University (approval number: ECSHU 2025-196). Post-traumatic osteoarthritis (PTOA) was established in 8-week-old male C57BL/6 mice via ACLT. Under anesthesia, the knee joint capsule was exposed in all animals. For the ACLT group, the anterior cruciate ligament was completely transected. Sham-operated controls received capsule exposure without ligament injury. Four weeks post-surgery, intra-articular injections were administered using a microsyringe every week: 10 μL of either PBS, LGG-EVs (1 × 10^10^ particles/mL), or W-LGG-EVs (1 × 10^10^ particles/mL). Five mice were used in each experimental group. Mice were euthanized 8 weeks after initial surgery for endpoint analysis. Knee joint samples were scanned using a high-resolution micro-CT system (Skyscan 1275, Bruker, Germany). The voxel size and exposure time per projection were 10 μm and 300 ms, respectively. Morphometric indices of the subchondral bone were analyzed, including bone volume fraction (BV/TV), the bone surface-to-volume ratio (BS/BV), trabecular thickness (Tb.Th), trabecular separation (Tb.Sp), trabecular pattern factor (Tb.Pf) and trabecular number (Tb.N). Knee joint specimens were sequentially fixed, decalcified, and embedded in puraffin. The sections were stained with hematoxylin and differentiated in 1% hydrochloric acid–ethanol. After rinsing with running water, some sections were counterstained with eosin to visualize the cytoplasm. Another portion was sequentially immersed in Fast Green and Safranin O staining solutions. Finally, all sections underwent dehydration and clearing simultaneously. The Osteoarthritis Research Society International (OARSI) histopathological scoring system was applied to evaluate the cartilage damage and loss. Sections were incubated in primary antibodies against COL II (Abcam, USA, 1:200), ACAN (Abcam, USA, 1:200), MMP13 (Abcam, USA, 1:200), ADAMTS5 (Abcam, USA, 1:200), and COL X (Abcam, USA, 1:250). For heat-induced antigen retrieval, the slides were immersed in EDTA buffer and incubated at 95 °C for 10 min. Hydrogen peroxide solution and BSA solution were sequentially added dropwise onto the sections to eliminate endogenous peroxidase activity and for blocking, respectively. A primary antibody at an appropriate concentration was applied to cover the samples and incubated at 4 °C overnight. The HRP-labeled secondary antibody was used to bind with the primary antibody. Stained sections were finally observed and analyzed.

### 2.11 MiRNA sequencing

RNA was purified from W-LGG-EV and processed for high-throughput sequencing following a previously described method. Small RNA sequencing libraries were prepared with the TruSeq Small RNA Sample Prep Kits (Illumina, San Diego, USA), and subsequent sequencing was carried out on an Illumina HiSeq platform.

### 2.12 Statistical analysis

Data are presented as mean ± standard deviation (SD). Statistical analyses were performed using GraphPad Prism 9. Differences between two groups were determined by Student's t-test, while comparisons among multiple groups were assessed by one-way ANOVA followed by Tukey's test. Significance levels were defined as follows: *p < 0.05, **p < 0.01, ***p < 0.001. All experiments were independently repeated at least three times.

## 3 Results

### 3.1 Natural LGG-EVs exhibit therapeutic potential for OA

LGG is one of the most extensively studied probiotics with a well-characterized genomic profile. It has been granted GRAS (Generally Recognized as Safe) status by the FDA. In addition, Lactobacillus species have a certain research foundation regarding chondrocyte function and the treatment of OA [16]. Studies have demonstrated that natural supplements primarily composed of LGG can reduce oxidative stress, attenuate adipogenesis, and promote the osteogenic differentiation of MSCs [24]. Studies have shown that oral administration of LGG can alleviate pain and inflammation associated with osteoarthritis [25,26]. Therefore, we cultured the probiotic LGG and isolated LGG-EVs using ultracentrifugation (Fig. 2A). TEM images indicated the isolated particles exhibited a bilayered spherical structure (Fig. 2B). The NTA data in Fig. 2C reveal a peak in particle size distribution at 153 nm, with no significant additional peaks. The concentration of these particles was 1.7 × 10^9^ particles/ml. These data confirm the successful extraction of LGG-EVs from the fermentation medium. Unlike mammalian EVs, BEVs lack specific markers, such as CD81, CD63 and TSG101 [27]. We were unable to verify the BEVs using specific marker proteins. The uncertainty of marker proteins is one of the major challenges in BEV research. Meanwhile, to further confirm that chondrocytes can internalize EVs, we explored the specific uptake mechanism. We treated cells with different inhibitors and observed the uptake of BEVs. Among them, Genistein showed the most significant inhibitory effect, indicating that the uptake of BEVs by chondrocytes is mainly mediated by caveolae-mediated endocytosis (Fig. S2A and B).

**Fig. 2:**
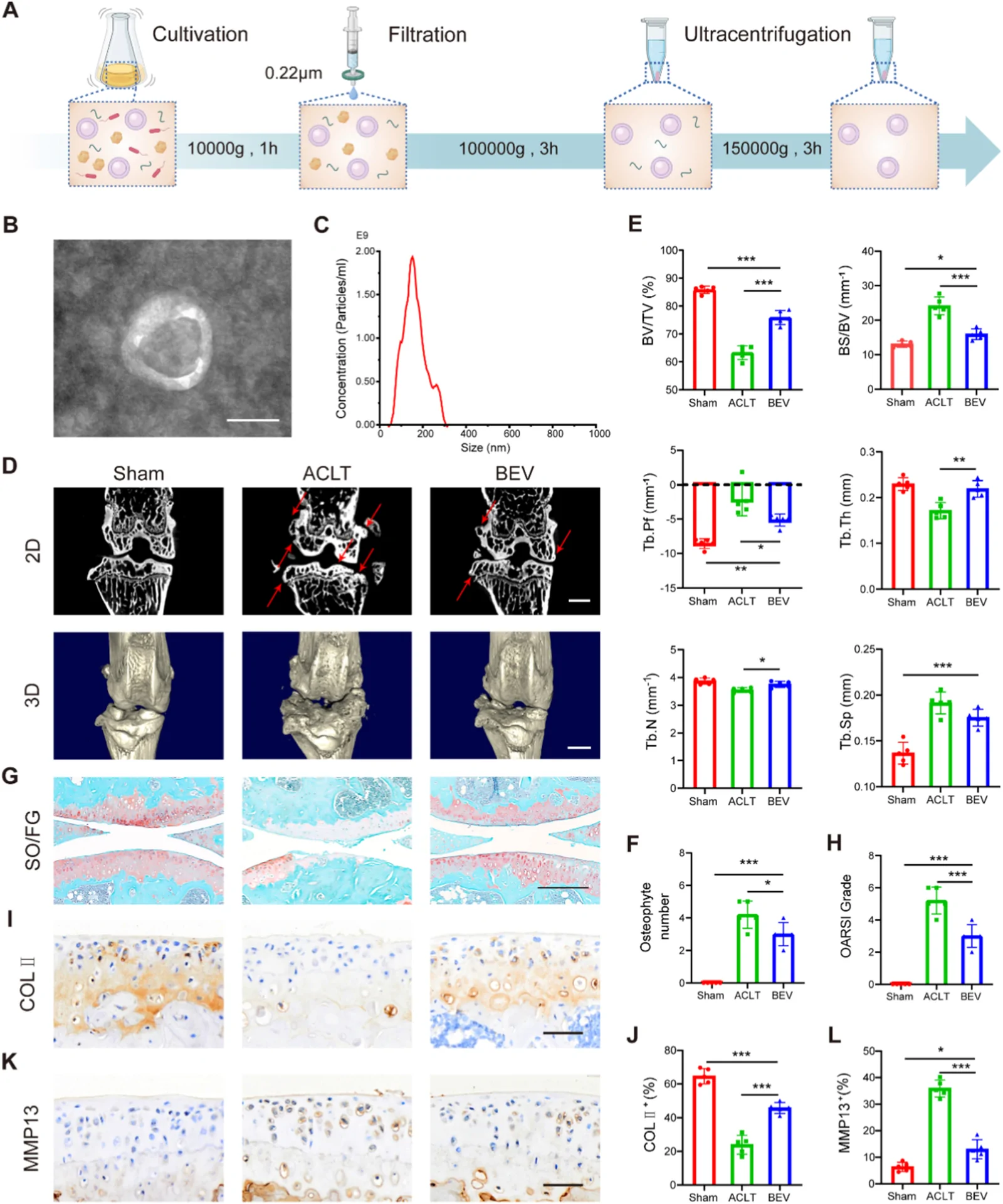
Isolation of natural bacterial vesicles and their therapeutic potential for OA. (A) Schematic diagram illustrating the extraction of LGG-EVs. (B) TEM of LGG-EVs. Scale bar: 100 nm. (C) Particle size distribution of LGG-EVs as determined by NTA. (D) Representative 2D micro-CT images depicting subchondral bone microstructure and osteophytes (red arrows). 3D reconstructed images visualizing joint surface morphology. Scale bar: 500 μm. (E) Parameters quantifying tibial subchondral bone microstructure. (F) Quantification of total osteophyte number per joint. (G) Representative images of SO&FG stained mouse knee joints 8 weeks after ACLT. Scale bar: 200 μm. (H) Osteoarthritis severity assessed using the OARSI scoring system. (I) Representative immunohistochemical staining images of COLII in tibial articular cartilage 8 weeks post-ACLT. Scale bar: 50 μm. (J) Quantitative analysis of COLII-positive area in articular cartilage. (K) Representative immunohistochemical staining images of MMP13 in tibial articular cartilage 8 weeks post-ACLT. Scale bar: 50 μm. (L) Quantitative analysis of MMP13-positive area in articular cartilage. Data are presented as mean ± SD; n = 5 per group. *p < 0.05, **p < 0.01, ***p < 0.001.

To evaluate the therapeutic potential of BEVs in vivo, we injected BEVs into the knee joints of OA mice. We evaluated the therapeutic effects of natural vesicles on the periarticular bone. Micro-CT was used to assess osteophytes and tibial subchondral bone. Representative 2D images showed narrowing of the joint space and abnormal osteophyte formation in the ACLT group (Fig. 2D and S3A). 3D reconstruction of the 2D images revealed an irregular bone surface with numerous and larger osteophytes in the ACLT group. In contrast, BEV treatment significantly ameliorated these pathological changes (Fig. 2D–F and S3B, C). Microstructural parameters of the tibial subchondral bone—including BV/TV, BS/BV, Tb.Pf, Tb.Th, Tb.N, and Tb.Sp—were analyzed (Fig. 2E). Compared to the ACLT group, BV/TV, Tb.Th, and Tb.N increased, while BS/BV and Tb.Pf decreased in the BEV group. These findings indicate that BEVs inhibit osteophyte formation. It is capable of ameliorating pathological lesions in the subchondral bone. In addition, H&E staining showed significant inflammatory cell infiltration and synovial thickening in the synovium of OA mice (Fig. S3D). BEV treatment alleviated inflammation and reduced the synovitis score (Fig. S3E). BEV effectively attenuated synovitis during OA progression. Furthermore, SO&FG staining and IHC were used to evaluate cartilage. Proteoglycan loss, cartilage damage, and erosion extending into the calcified zone were observed in the OA group. Intra-articular injection of BEVs showed a protective effect on the cartilage. BEV treatment significantly improved cartilage structure and morphology, with a more intact cartilage surface and a reduced OARSI score (Fig. 2G and H). Meanwhile, the expression of COLⅡin OA mouse cartilage was reduced (Fig. 2I and J), and MMP13 expression was increased (Fig. 2K and L). BEV treatment significantly reversed these effects. These data provide a compelling body of evidence supporting the therapeutic potential of BEVs in the treatment of OA.

### 3.2 Preparation and characterization of engineered W-LGG-EVs

While natural LGG-EVs exhibited therapeutic potential for OA, their efficacy remained limited. Due to the dense cartilage matrix and the rapid metabolism of synovial fluid, LGG-EVs have difficulty remaining in the joint cavity for extended periods and penetrating into the deep layers of cartilage. These factors limit the therapeutic efficacy of LGG-EVs [28,29]. Therefore, we engineered LGG-EVs according to the strategy in Fig. 3A. The type II collagen-specific targeting peptide was covalently conjugated to the surface of BEVs to generate engineered W-LGG-EVs, which exhibit enhanced adhesion within the joint cavity and targeted binding capacity toward cartilage tissue. TEM images confirmed that the morphology of the modified W-BEVs remained as typical spherical bilayer vesicles (Fig. 3B). NTA revealed a predominant particle size distribution peak at 160 nm. The concentration was measured to be 1.1 × 10^9^ particles/mL (Fig. 3C). Following successful surface modification, we assessed the surface charge of the vesicles. LGG-BEVs presented an average zeta potential of −16.29 ± 1.22 mV, while the average zeta potential of W-BEVs was 8.00 ± 2.29 mV (Fig. 3D). To confirm the successful modification of W-BEV, the WYRGRL peptide was conjugated with FITC. Nano-flow cytometric analysis revealed abundant fluorescent signals on W-BEV, demonstrating that the cartilage-targeting peptide had been effectively anchored to the BEV surface, and the binding efficiency of the peptide was determined to be 73.5% (Fig. 3E). These results demonstrate the successful surface functionalization of BEVs with the WYRGRL peptide. This modification preserved the fundamental vesicular morphology while effectively altering the particle size and surface charge.

**Fig. 3:**
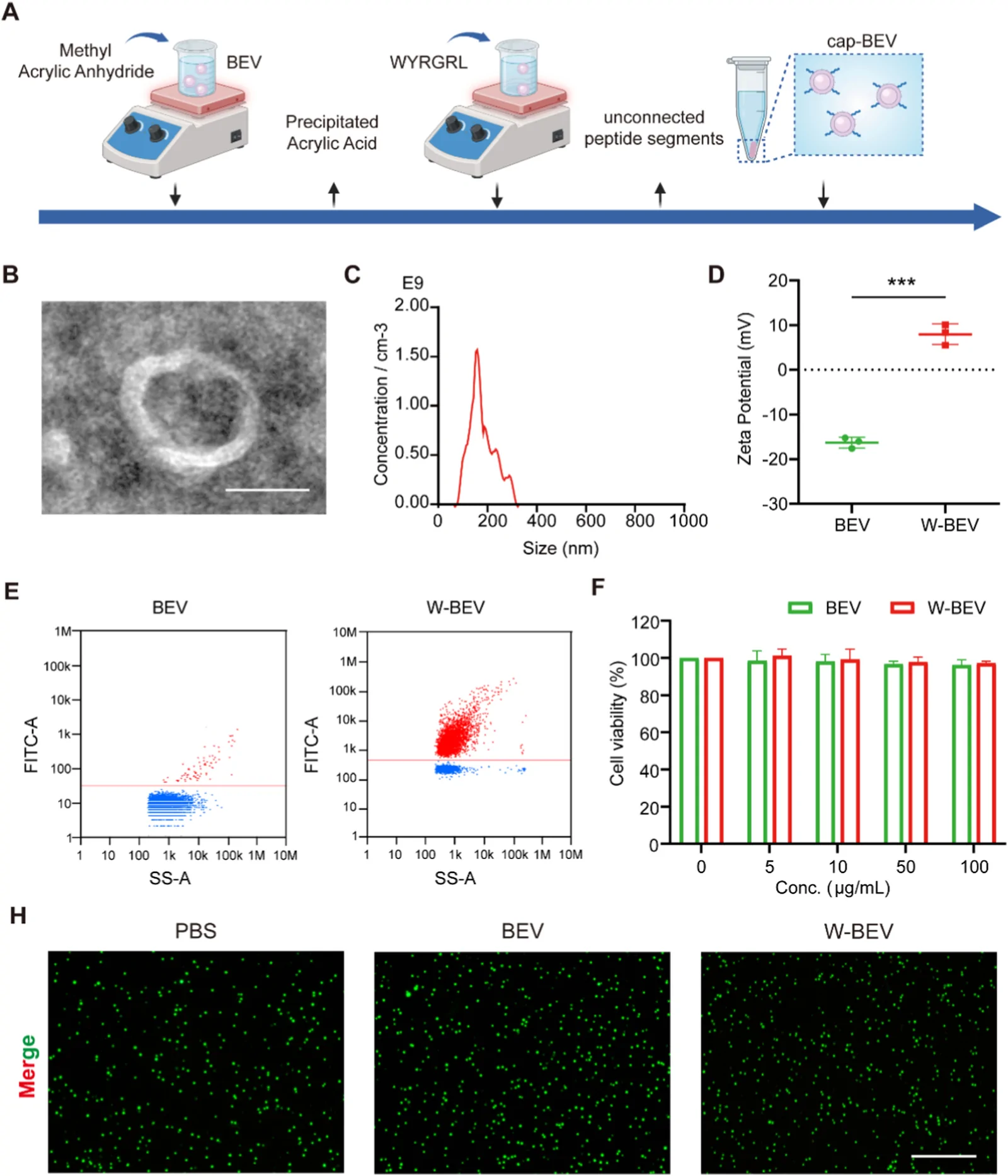
Design and characterization of W-BEVs. (A) Schematic illustration of W-LGG-EV construction. (B) TEM of LGG-EVs. Scale bar = 100 nm. (C) Particle size distribution of W-LGG-EVs determined by NTA. (D) Surface potential of BEVs and W-BEVs. (E) Nanoflow cytometry (NFC) analysis of the WYRGRL modification rate of BEVs. (F) Cytotoxicity of LGG-EVs and W-LGG-EVs in vitro. (G) Dead/Live (D/L) staining of chondrocytes treated with PBS, LGG-EVs, and W-LGG-EVs. Scale bar = 500 nm. Data are presented as mean ± SD; n = 3. *p < 0.05, **p < 0.01, ***p < 0.001.

It has been previously demonstrated that BEVs derived from either non-pathogenic or detoxified bacteria exhibit favorable biosafety [30]. We further evaluated the safety of engineered BEVs to determine their potential for subsequent applications. Firstly, we incubated PKH-labeled W-BEVs with cells to assess cellular uptake. Fluorescence imaging revealed substantial internalization of W-BEVs by the cells (Fig. S4A). W-BEVs do not impede cellular uptake, providing an opportunity for the delivery of bioactive molecules encapsulated within the vesicles [31]. We further assessed the cellular compatibility of W-BEVs. Live/dead cell staining and CCK-8 assay showed that BEVs and W-BEVs exhibited no apparent cytotoxic effects (Fig. 3F and G). To evaluate systemic safety, we administered BEVs and W-BEVs and monitored histopathological changes in major organs. No apparent pathological changes were observed in the heart, liver, spleen, lung, or kidney (Fig. S4B). In summary, we successfully constructed engineered BEVs that exhibit no apparent toxicity.

### 3.3 Enhanced cartilage-targeting efficacy of W-LGG-EVs

The cartilage-targeting capability of W-BEVs was validated both in vitro and in vivo. We first evaluated the retention of W-BEVs within the joint cavity. DiR-labeled BEVs and W-BEVs were administered into the knee joints and imaged (Fig. 4A). The fluorescence signal intensity naturally decreased over time in both groups. However, the signal decay was slower in the W-BEVs group. BEVs were retained in the joint cavity for only three days, whereas W-BEVs persisted for 7-10 days and were completely cleared by day 14. Targeted modification significantly slowed the metabolism of the vesicles (Fig. 4B). After the second day post-injection, the signal intensity of W-BEVs was higher than that of BEVs (Fig. 4D). Three days after injection, we dissected the mice and collected major organs. The heart, liver, spleen, lungs, kidneys, and knee joints were imaged using the IVIS Spectrum system (Fig. 4A). The liver in the BEV group exhibited strong fluorescence signals, indicating substantial accumulation of BEVs. In contrast, no discernible fluorescence was observed in the W-BEV group (Fig. 4C and S5A). The targeting peptide significantly enhanced the retention of W-BEVs within the joint cavity and reduced their distribution to other organs.

**Fig. 4:**
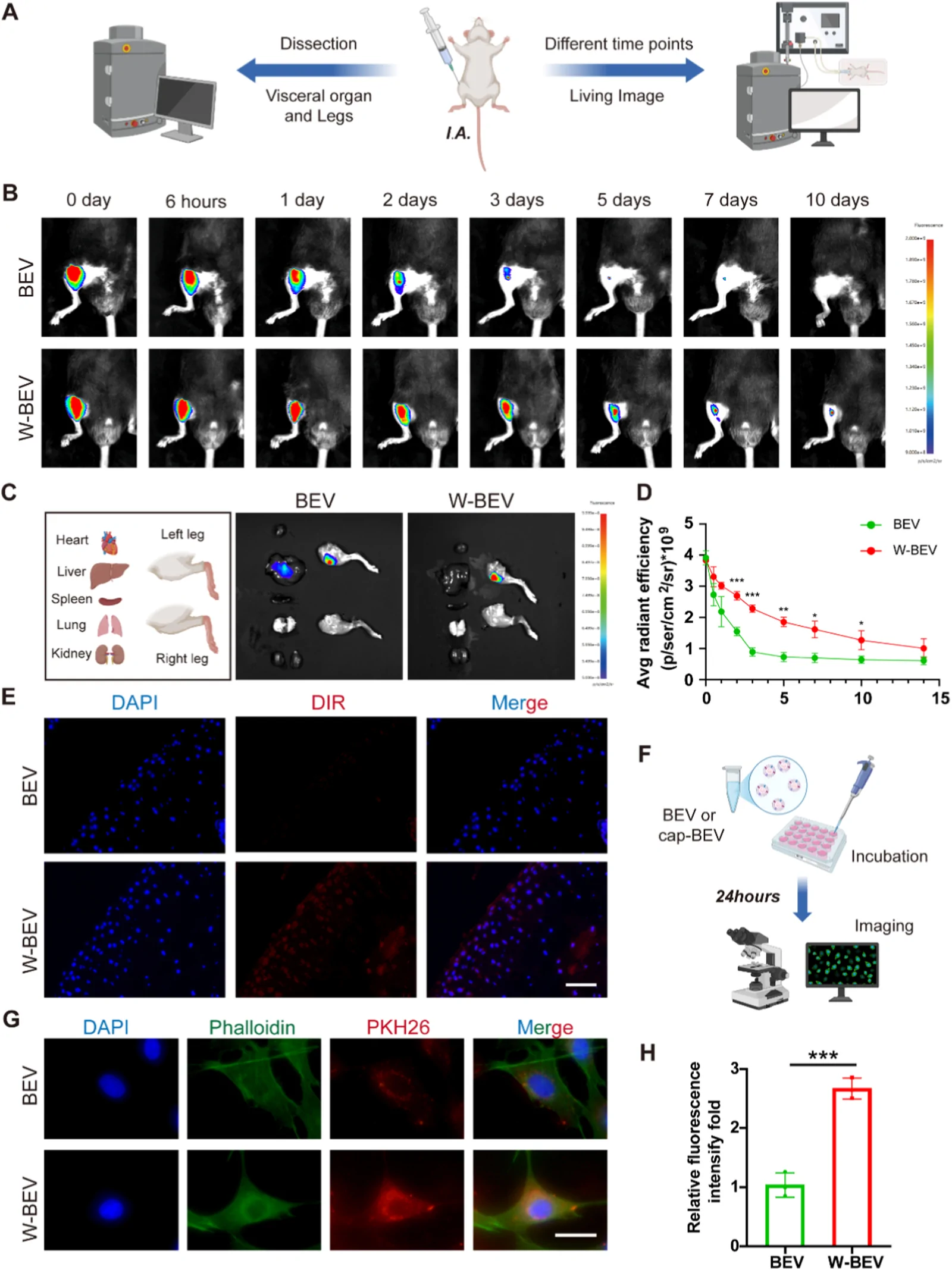
Enhanced chondrocyte-targeting efficacy of W-LGG-EVs in vivo and in vitro. (A) Schematic diagram of the experimental procedure for validating in vivo chondrocyte-targeting efficacy. (B) Fluorescence imaging of rat knee joints at 6 h, 1 day, 2 days, 3 days, 5 days, 7 days, and 10 days post-intra-articular injection of DIR-labeled BEVs and W-BEVs using an IVIS Spectrum imaging system. (C) Fluorescence intensity in major organs (heart, liver, spleen, lung, kidney) and knee joints of mice 48 h post-intra-articular injection of DIR-labeled BEVs and W-BEVs. (D) Quantitative analysis of fluorescence intensity from the groups. (E) Representative images of mouse knee joint sections following intra-articular injection of DIR-labeled BEVs and W-BEVs. Scale bar = 50 μm. (F) Schematic diagram of the experimental procedure for validating in vitro chondrocyte-targeting efficacy. (G) Fluorescence images of chondrocytes incubated with PKH26-labeled BEVs and W-BEVs for 24 h. Scale bar = 25 μm. (H) Semi-quantitative analysis of relative fluorescence intensity of BEVs and W-BEVs. Data are presented as mean ± SD; n = 3. *p < 0.05, **p < 0.01, ***p < 0.001.

Subsequently, We demonstrated the cartilage-targeting adhesion and penetration capability of W-BEVs in vivo. Three days after injection, joint sections revealed that the red fluorescence signal in the BEV group was mainly localized to the superficial layer of the cartilage (Fig. 4E). In contrast, The W-BEV group exhibited stronger red fluorescence. Distinct red fluorescence signals were also observed within the deeper layers of the cartilage. The active movement and homing mechanisms of vesicles determine their penetration capability [32]. However, modification with WYRGRL enhanced the adhesion of BEVs and their ability to penetrate the cartilage. In summary, W-BEVs exhibit strong cartilage-targeting capability in vivo. It can remain in the joint cavity for an extended period and adhere to the cartilage.

We further incubated BEVs with chondrocytes or fibroblasts (Fig. 4F). After 24 h, chondrocytes treated with W-BEVs exhibited bright red punctate fluorescence. The fluorescence intensity was higher than that observed in BEVs group (Fig. 4G). Compared to BEVs, W-BEVs promoted the uptake of vesicles by chondrocytes. In fibroblasts, both BEV and W-BEV groups exhibited similar levels of red fluorescence (Fig. S5B and C). In the W-BEV group, chondrocyte uptake was significantly enhanced. But the uptake levels in fibroblasts were similar across all groups (Fig. 4H). Compared to BEVs, W-BEVs exhibit a stronger cartilage-targeting effect. W-BEVs can more effectively bind to the negatively charged cartilage tissue, thereby enhancing retention in the complex and dynamic environment.

### 3.4 W-LGG-EVs promote extracellular matrix synthesis in vitro

OA is characterized by impaired chondrocyte function and an inability to self-repair cartilage lesions, leading to dysregulated ECM composition [33]. Consequently, preserving normal ECM secretion is critical for cartilage homeostasis. To determine whether W-BEVs exhibit enhanced therapeutic effects, we first treated cells with BEVs or W-BEVs for 24 h. Subsequently, the chondrocytes were maintained for an additional 24 h in fresh medium supplemented with IL-1β to simulate the clearance dynamics of synovial fluid in vivo (Fig. 5A). IL-1β treatment of chondrocytes in vitro leads to downregulation of anabolic activity and upregulation of catabolic processes, effectively mimicking the pathological features of OA chondrocytes [34].

**Fig. 5:**
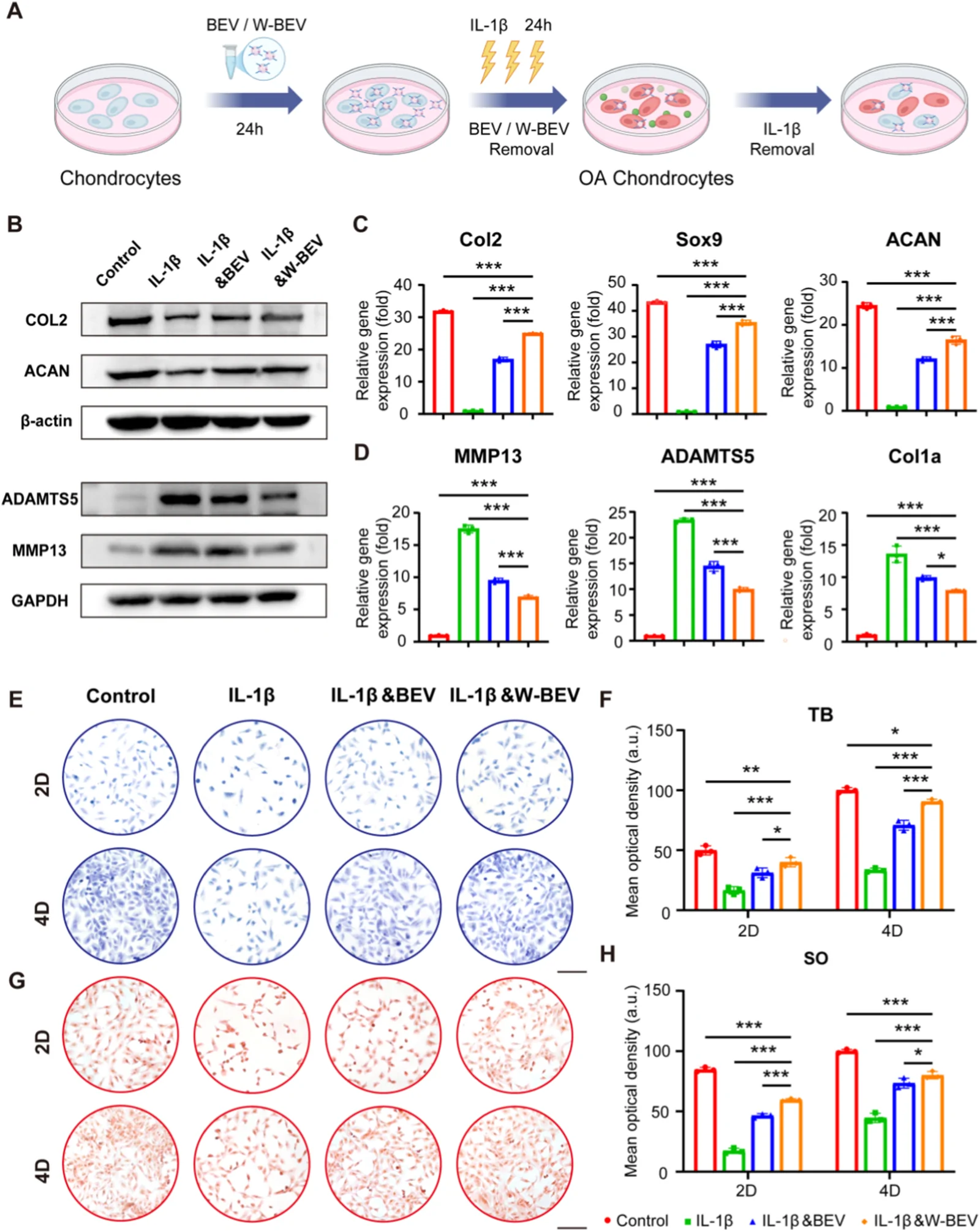
W-LGG-EVs promote ECM synthesis and accelerate cartilage matrix regeneration in OA chondrocytes in vitro. (A) Schematic diagram of the in vitro experimental design for evaluating the sustained therapeutic effects of W-BEVs. (B) Representative Western blot analysis of anabolic and catabolic protein levels in IL-1β-induced OA chondrocytes treated with W-BEVs versus BEVs. (C, D) Expression of specific matrix-related genes assessed by RT-qPCR at indicated time points. (E) Representative Safranin O-stained images of OA chondrocytes incubated with different treatments at day 2 and day 4. Scale bar = 150 μm. (F) Semi-quantitative analysis of SO staining intensity across groups. (G) Representative TB-stained images of OA chondrocytes incubated with different treatments at day 2 and day 4. Scale bar = 150 μm. (H) Semi-quantitative analysis of TB staining intensity across groups. Data are presented as mean ± SD; n = 3. *p < 0.05, **p < 0.01, ***p < 0.001.

To determine the impact of W-BEV on ECM synthesis in OA chondrocytes, we analyzed key protein and mRNA expression profiles. As shown by Western blot analysis, catabolic factors MMP13 and ADAMTS5 were upregulated while the anabolic factor COL2 and ACAN was downregulated. W-BEVs exerted a more effective reversal of these expression changes compared to BEVs (Fig. 5B). Results from RT-qPCR confirmed that at the mRNA level, W-BEV-treated OA chondrocytes exhibited upregulation of matrix-related genes along with downregulation of matrix-degrading genes and pro-inflammatory genes (Fig. 5C and D, and S6). Furthermore, the expression levels of COL II and MMP13, as assessed by immunofluorescence, showed analogous alterations in the BEV and W-BEV treatment groups (Fig. S7A and B). To further determine the effects of W-BEVs on OA chondrocytes, TB and SO staining were performed on chondrocytes on days 2 and 4. As illustrated in Fig. 5E,F,G,H, cartilage-associated matrix staining revealed a consistent trend across groups: W-BEVs > BEVs > OA group. By enhancing the uptake rate of chondrocytes, W-BEVs more effectively alleviated the proteoglycan loss induced in OA chondrocytes (Fig. 5F–H). W-BEVs significantly enhanced the quality of regenerated cartilage matrix in OA chondrocytes. As shown in Fig. S8A, the migration area in the W-BEV group was substantially larger compared to other groups. Quantitative analysis of the residual wound area revealed the lowest values in the W-BEV group, indicating that W-BEVs enhanced the migratory capacity of chondrocytes (Fig. S8B). We next explored the effect of W-BEV on cellular proliferation. The relative proliferative capacity was assessed using the EdU incorporation assay. The W-BEV-treated group exhibited a significantly higher relative proliferation rate compared with the other groups (Fig. S9A and B). WYRGRL enhanced the targeting ability of BEVs toward type II collagen and increased the uptake of BEVs by chondrocytes. Therefore, even under in vitro conditions simulating synovial fluid clearance, cells can continue to utilize the absorbed probiotic vesicles for an extended period, thereby enhancing their therapeutic effects on OA chondrocytes.

### 3.5 W-LGG-EVs effectively alleviate OA progression in vivo

We utilized a mouse OA model established by ACLT to evaluate whether W-BEVs could achieve superior therapeutic effects in vivo (Fig. 6A). The experimental groups consisted of: PBS, BEVs, W-BEVs, and sham-operated group. Joints from each group were characterized. We first performed micro-CT reconstruction and examined the joint images of each group to evaluate the overall morphology of the mouse joints (Fig. 6B). Severe deformation of the subchondral bone was observed in the PBS group. Compared to the BEV group, the overall morphology in the W-BEV group was markedly improved at 8 weeks, more closely resembling that of the sham group. Osteophytes are an important indicator of OA severity. Except for the sham-operated group, osteophytes were present in all other groups (Fig. 6C). The PBS group exhibited the most pronounced and numerous osteophytes, while the W-BEV group exhibited the smallest and fewest osteophytes. W-BEVs enhanced the therapeutic effect of BEVs (see Fig. 7).

**Fig. 6:**
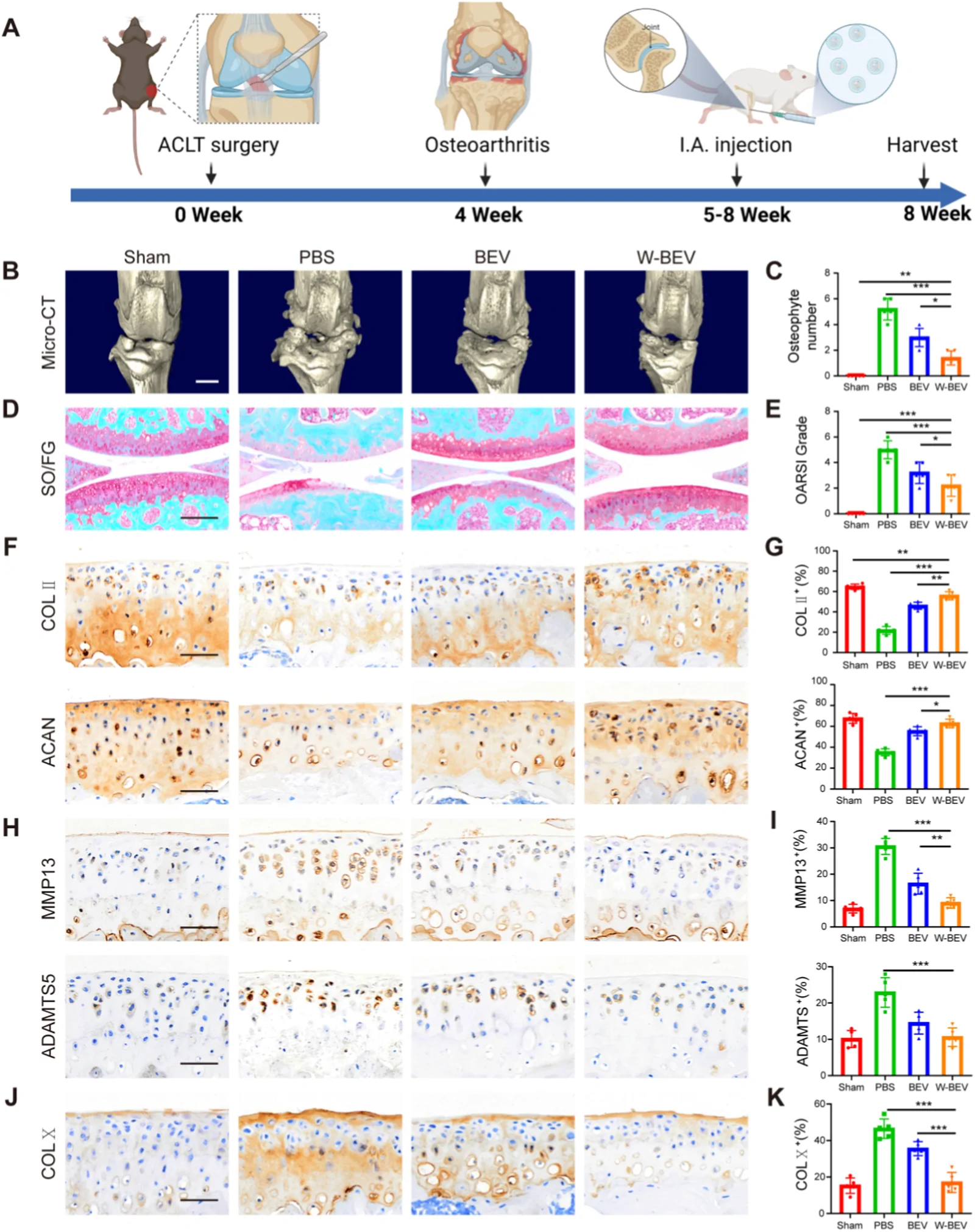
Therapeutic efficacy of W-LGG-EVs against OA in vivo. (A) Schematic diagram of OA amelioration in mouse model. (B) 3D reconstructed micro-CT images depicting joint surface morphology. Scale bar: 500 μm. (C) Quantification of total osteophyte number per joint. (D) Representative SO&FG stained sections of mouse knee joints 8 weeks post-ACLT. (E) Osteoarthritis severity assessed using OARSI scoring system. (F-K) Representative immunohistochemical staining images of COL2, ACAN, MMP13, ADAMTS5, and COLX in tibial articular cartilage 8 weeks post-ACLT. Quantitative analysis of positively stained areas for respective biomarkers.Scale bar: 50 μm. Data presented as mean ± SD; n = 5 per group. *p < 0.05, **p < 0.01, ***p < 0.001.

To assess the structure of articular cartilage and the quality of proteoglycans, HE and SO&FG staining were performed at 8 weeks (Fig. 6D and S10A). In the sham and W-BEV group, the articular cartilage exhibited uniform thickness and a smooth surface. And the chondrocytes were orderly arranged. In contrast, the cartilage matrix was thin and uneven in the PBS group. Portions of the subchondral bone surface were exposed due to the absence of cartilage coverage. In the BEV group, cartilage thickness was improved, but the edges remained uneven. Significant differences were observed among the groups in cartilage structure, matrix quality, and the integrity of the tidemark (Fig. 6E and S10B). The specific targeting peptide facilitated higher accumulation of BEVs on the cartilage surface. W-BEVs significantly enhanced the therapeutic effects on chondrocytes and promoted the repair of articular cartilage.

Histological staining results showed that the cartilage matrix quality was superior in the vesicle-treated groups. Therefore, we further analyzed the effect of W-BEV on articular cartilage via IHC. As shown in Figs. 6F and 5G, the addition of W-BEV more effectively improved cartilage matrix quality compared to BEV. COLⅡ and ACAN, major components of healthy hyaline cartilage, were significantly expressed in the W-BEV group. MMP13 and ADAMTS5 are key matrix-degrading enzymes during OA progression. Compared to the PBS and BEV groups, the W-BEV group exhibited lower expression levels of MMP13 and ADAMTS5. The low expression of these degrading enzymes confirmed that the engineered vesicles based on LGG-EV further delayed OA progression (Fig. 6H and I). COLⅩ, secreted by hypertrophic chondrocytes, shows elevated expression in OA cartilage [3]. In the treatment groups, COLⅩ expression followed a similar trend to MMP13 expression. W-BEV effectively reduced COLⅩ secretion (Fig. 6J and K). Overall, W-BEV treatment yielded the best overall outcome, closest to the native group. This suggests that cartilage-specific targeting and the intrinsic properties of the probiotic vesicles exert a synergistic effect on LGG-EV therapy for OA. W-BEV holds potential clinical translational value for preserving and treating articular cartilage structure in OA patients.

### 3.6 Analysis of the microRNA expression profile in W-LGG-EVs

BEVs can deliver bioactive molecules to articular cartilage [35]. Probiotics EVs serve as a promising nanodrug delivery platform capable of delivering bacterial endogenous cargo, such as miRNAs and proteins, to modulate host cellular physiological and pathological processes. The protein composition and functionality of LGG-BEVs have been initially characterized. A comprehensive proteomic study of BEVs produced by LGG across various growth phases has been reported [36]. The identified proteins are predominantly cytoplasmic membrane-associated and cytoplasmic proteins, and their functional classification implicates pathways related to environmental information processing, genetic information processing, and carbohydrate metabolism. Furthermore, the LGG-EV proteome includes several enzymes possessing immunomodulatory properties, such as aminopeptidases and aminoacyl-tRNA synthetases. At the functional level, LGG-EVs are capable of triggering host immune responses via the TLR2/CD14 and TLR6/2 signaling axes, and they stimulate macrophages to secrete TNFα, IL-6, and IL-10—key immunoregulatory cytokines. Collectively, these observations point to the possible therapeutic utility of BEVs in OA [37].

The biological activity of extracellular vesicles has also been linked to miRNAs [38]. Furthermore, it has been reported that LGG-derived BEVs are capable of delivering miR-21-5p, thereby promoting wound healing [39]. Therefore, to elucidate the functional miRNAs within W-LGG-EVs, next-generation sequencing (NGS) was performed. Furthermore, studies have found that physicochemical modifications do not significantly alter the cargo content of vesicles [31]. The expression profiles of the top 20 miRNAs in W-BEVs are shown in Fig. 7A. Importantly, miR-122 [40,41], miR-27 [42], miR-21 [43], and miR-199 [44] have been shown to treat OA. Although the specific regulatory mechanisms by which miR-486 [45], miR-451 [46], and miR-185 [47] influence chondrocyte function remain unclear, they are enriched in many exosomes or botanical extracts that can ameliorate OA. MNDR enrichment analysis of the highly expressed miRNAs (Fig. 7B) revealed that W-LGG-EVs are highly enriched in immune and inflammatory responses, and are also closely associated with musculoskeletal diseases. This indicates a direct potential association between W-LGG-EVs and OA. Subsequently, we conducted Gene Ontology (GO) and Kyoto Encyclopedia of Genes and Genomes (KEGG) enrichment analyses on the top 20 miRNAs to explore their potential involvement in biological processes and signaling pathways. As shown in Fig. 7C, KEGG analysis indicated that these target genes are primarily enriched in the Wnt signaling pathway, TGF-β signaling pathway, and HIF-1 signaling pathway. The miRNAs within W-LGG-EVs can broadly regulate the cartilage microenvironment, with the significantly enriched signaling pathways highly concentrated in inflammatory responses, immune regulation, and cellular stress metabolic mechanisms. GO analysis revealed (Fig. 7D) that the top 20 miRNAs participate in biological processes (BPs) such as cytokine response, DNA damage response, protein glycosylation, DNA methylation, cell cycle, and cell proliferation. They also influence cellular components (CCs), including the nuclear replication fork, autophagosome, Golgi stack, and nuclear chromosome. Furthermore, the molecular functions (MFs) from GO analysis indicated that the top 20 miRNAs regulate protein receptor binding, protein activity, kinase activity, DNA binding, and mitochondrial metabolism.

**Fig. 7:**
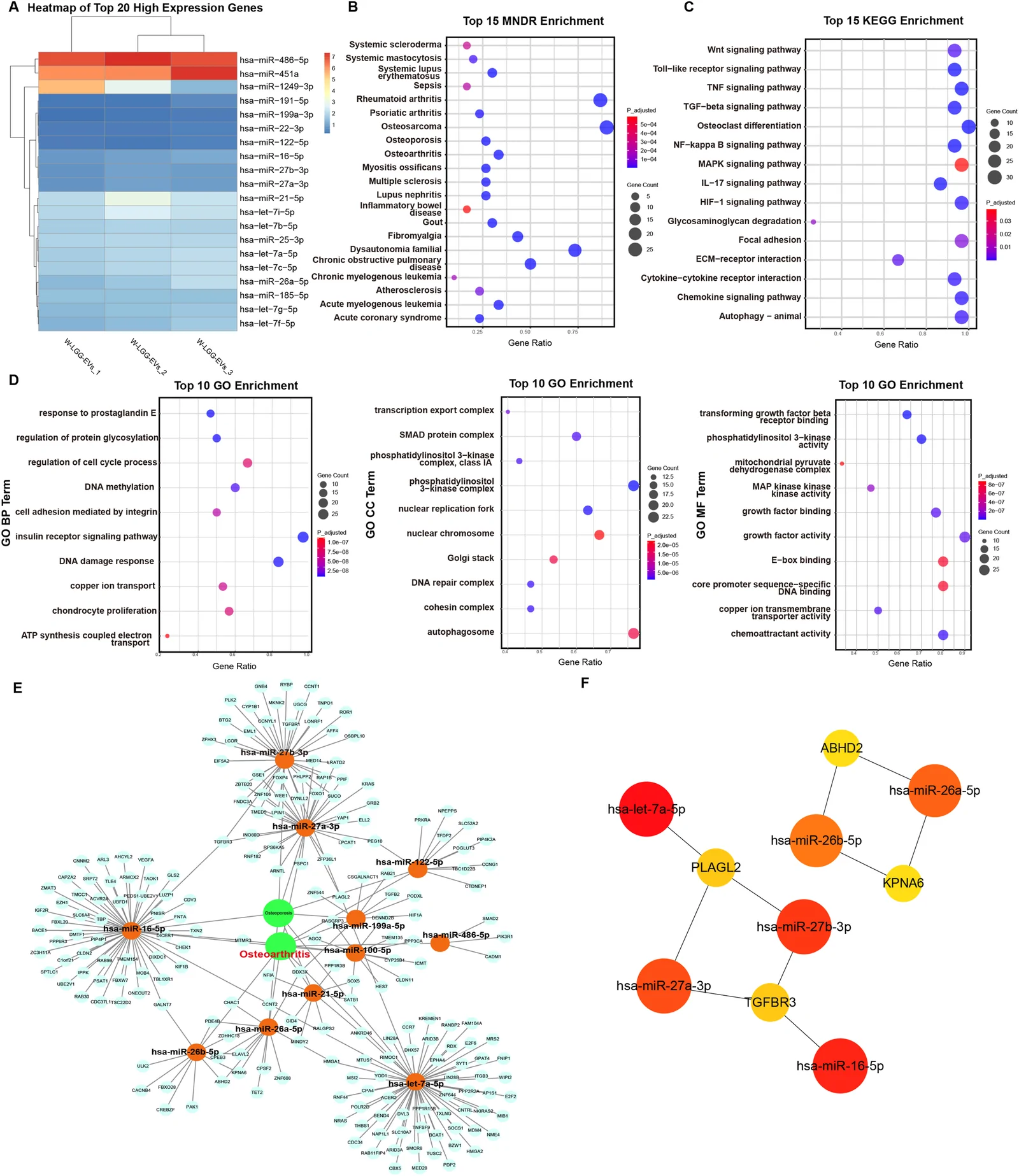
miRNA Profiling of W-LGG-EVs. (A) Heatmap analysis of the top 20 significantly expressed miRNAs in W-LGG-EVs. (B) miRNA Network Database Resource (MNDR) analysis of the top 20 significantly expressed miRNAs. (C) KEGG pathway enrichment analysis of the top 20 significantly expressed miRNAs. (D) Gene Ontology (GO) analysis of the top 20 miRNAs, including biological processes (BP), cellular components (CC), and molecular functions (MF). (E) Schematic diagram of predicted target genes for five selected miRNAs among the top 20 significantly enriched miRNAs in W-LGG-EVs. (F) Subnetwork of osteoarthritis (OA)-related key genes.

Subsequently, the interaction networks and gene clusters of these miRNA target genes were visualized using Cytoscape software (Fig. 7E). MiR-27b-3p, miR-26b-5p, and miR-16-5p emerged as key mediators in the pathogenesis of OA. Fig. 7F shows that they influence synovial inflammatory responses and chondrocyte metabolism by regulating the TGF-β pathway, TLR3/COL10A1, and SMAD3, respectively. Overall, the miRNAs within W-LGG-EVs and their target genes demonstrate a broad regulatory network in OA, exhibiting potential value as biomarkers and therapeutic targets.

## 4 Discussion

The unique structure of joints dictates that articular cartilage exists in an avascular state. Consequently, articular cartilage is relatively unresponsive to systemic drug administration, and direct intra-articular injection is the standard clinical approach [17,48]. Intra-articular injection therapy can be used to control the acute phase or for chronic management to mitigate OA progression. Currently, commonly used intra-articular injectable agents mainly include corticosteroids, hyaluronic acid, NSAIDs, and bisphosphonates [49,50]. However, these drugs often come with significant side effects and show limited therapeutic efficacy in clinical practice. Therefore, novel agents for intra-articular injection are essential. Various biomaterials, such as hydrogels, nanoparticles, and liposomes, have been investigated for delivering drugs into the joint cavity. An ideal clinical drug should not only leave the joint cavity rapidly but also be suitable for large-scale industrial production.

The GM demonstrates a close association with the pathogenesis and progression of OA [51]. The direct communication is referred to as the “gut-joint axis” [52,53]. Several probiotics, such as *Lactobacillus casei* [54], *Lactobacillus rhamnosus* [25], *Lactobacillus acidophilus* [55], and *Lactobacillus plantarum* [56], have been reported to improve joint health by regulating intestinal barrier integrity and immunity. Studies have demonstrated their potential to alleviate disease progression and reduce pain [57,58]. Current research mainly focuses on the oral administration of probiotics, because probiotics regulate OA through various bioactive molecules rather than through direct bacterial translocation [35]. Therefore, extracting bioactive molecules from probiotics and converting them into intra-articularly injectable drugs holds promising clinical application prospects. BEVs, as spherical membranous structures secreted by GM, can encapsulate and deliver a variety of bacterial bioactive molecules, playing a crucial messenger role in intercellular communication [59]. Studies have shown that BEVs released by probiotics can also regulate metabolic functions in skeletal and synovial structures to control disease progression [23,60]. This provides a new approach for OA treatment. Since GM can mediate gut–joint interactions through the secretion of various factors, it is possible to harness specific probiotic-derived products for OA therapy [61]. Vesicles derived from *Lactobacillus rhamnosus* have been shown to control joint inflammatory and delay OA progression [16]. Therefore, we propose that BEVs serve as a novel bridge in the gut–joint axis, mediating the connection between the gut and OA [35]. Compared to mixed microbiota derived from feces, which have complex compositions and large batch-to-batch variability, we chose to first use standard strains to explore the potential of probiotic BEVs for OA. This ensures the stability of the extracted BEVs. Although direct studies on LGG-EVs in OA are still lacking, their relevant composition and functions suggest therapeutic potential. The lipoteichoic acid (LTA) carried by LGG-EVs has been shown to modulate immune responses through the TLR2 pathway, and TLR2 signaling plays an important role in regulating OA inflammation. Moreover, multiple proteins in the LGG-EVs can interact with TNFα and IL-6 signaling pathways. Given that OA is a joint disease characterized by chronic low-grade inflammation, LGG-EVs hold significant promise for delaying OA progression [36].

But their retention within the joint cavity is limited. An ideal intra-articular injectable should exhibit prolonged retention within the cartilage layer, penetrate the cartilage matrix, and undergo endocytosis by chondrocytes [62]. BEVs can be engineered to confer novel properties meeting the requirements for ideal intra-articular therapeutics. Engineered BEVs produced by synthetic biology tools can overcome the limitations of current OA therapies [[52], [53], [54]]. In our previous research, we highlighted engineering strategies for modifying BEVs to treat degenerative bone diseases, including direct genetic modification of parent strains to produce therapeutic BEVs, and functionalization of isolated BEVs [63]. We also validated the application of engineered BEVs for Osteoporosis treatment [63,64]. Therefore, we attempted to further modify LGG-EVs to enhance their therapeutic efficacy against OA.

In this study, we constructed a novel engineered LGG-EV for OA therapy. Given the rapid proliferation of bacteria and mature cultivation techniques, BEV-based therapeutics can overcome the large-scale production challenges faced by mammalian and other synthetic nanomaterials [11]. As current genetic engineering methods for LGG-EVs remain largely unexplored [64], LGG-EVs were functionalized with cartilage-targeting properties through the application of conventional physicochemical methods. Subsequently, we modified the BEVs with WYRGRL to enhance their cartilage-targeting capability. WYRGRL is a short peptide composed of six amino acids. It can specifically bind to the alpha 1 chain of type II collagen in articular cartilage. WYRGRL has been demonstrated in multiple studies to possess excellent cartilage-targeting properties [65,66], with its targeting ability in mouse joints reported to be enhanced by up to 72-fold [67]. Our results confirmed that the engineered modification did not affect the inherent morphology of the BEVs. W-BEVs exhibited good biocompatibility and represent a safe strategy for OA therapy. Concurrently, the targeting modification prolonged the retention time of BEVs within the joint cavity and enhanced their uptake by chondrocytes. While the retention-enhancing effect conferred by targeted modification is mild, it is sufficient to meet clinical needs. In clinical practice, intra-articular drugs such as hyaluronic acid are often administered once a week with multiple injections [68]. The metabolic turnover of synovial fluid results in near-total depletion of conventional drugs within 2-3 days [69]. W-BEV minimizes the drug-free interval, ensuring that the intra-articular drug concentration remains at therapeutic levels for a prolonged time, lasting until the next dose. The best therapeutic outcomes of W-BEV are achieved with repeated administrations. We plan to further refine our modification approach, aiming to develop agents with prolonged efficacy and reduced injection frequency.

Based on our study, we modified probiotic-derived vesicles for the treatment of OA for the first time. Numerous studies have reported that Lactobacillus species and their related products possess therapeutic potential for OA [35,55,70]. As important secretory products of probiotics, BEVs may represent a promising strategy for cartilage regeneration and OA treatment. Our study further provides in-depth validation of the hypothesis. Cartilage ECM damage is a hallmark of OA. The ability of chondrocytes to secrete ECM becomes dysregulated in the pathological process. The rate of catabolism exceeds that of anabolism, thereby disrupting the homeostatic balance of the ECM [71]. We confirmed that BEVs can restore the impaired function of chondrocytes, thereby controlling the progression of cartilage damage in OA. Our study also has limitations. Gut bacteria can collectively regulate the progression of OA [15]. The probiotics we selected are relatively simple. In subsequent studies, we will further compare the gut bacteria of OA patients and attempt to screen and isolate more targeted specific strains and vesicles from the pathological environment. Research suggests that subchondral bone abnormalities are also significant factors in OA development [72,73]. Whether BEVs can penetrate into the subchondral bone and exert regulatory effects on the subchondral bone microenvironment remains unclear. Additionally, before translating this strategy into clinical applications, further studies are needed to evaluate the efficacy and safety of W-BEVs in large-animal OA models.

## 5 Conclusion

In this study, we engineered natural probiotic bacterial extracellular vesicles with cartilage-targeting modifications. This cell-free therapy for osteoarthritis effectively maintains the metabolic homeostasis of the cartilage matrix. Engineered BEVs can rapidly adhere to the articular cartilage surface and effectively penetrate the negatively charged cartilage matrix to reach the deeper layers. BEVs deliver bioactive molecules such as miRNAs to chondrocytes, thereby maintaining cartilage metabolic homeostasis. From a future clinical perspective, we substantiate the crucial role of BEVs within the gut–joint axis. This study provides a new avenue for exploring the link between the GM and OA. Bacterial products, particularly BEVs, possess potential for treating degenerative joint diseases. The engineered W-BEVs can resist clearance by synovial fluid, thereby maintaining an effective drug concentration for an extended period. The newly developed W-BEVs exhibit significant potential for OA treatment in clinical practice. Besides, BEVs can also serve as carriers for delivering various drugs, enabling targeted treatment of OA and other degenerative diseases. These findings offers a novel drug delivery strategy for precision medicine, which support the further development of BEVs-based platforms for targeted drug delivey systems.

## CRediT authorship contribution statement

**Luyu Niu:** Writing – original draft, Investigation, Formal analysis. **Wanzhuo Chen:** Writing – original draft, Methodology, Investigation. **Tong Liu:** Methodology, Investigation, Data curation. **Xiaoyi Liang:** Methodology, Investigation, Data curation. **Peiran Song:** Investigation, Data curation. **Han Liu:** Data curation, Conceptualization. **Teng Su:** Methodology, Investigation. **Xiao Chen:** Resources, Formal analysis. **Zhongtang Liu:** Writing – review & editing, Validation, Project administration, Conceptualization. **Yingying Jing:** Writing – review & editing, Validation, Project administration. **Jin Cui:** Writing – review & editing, Project administration, Funding acquisition. **Jiacan Su:** Resources, Project administration, Funding acquisition, Conceptualization.

## Ethics approval and consent to participate

All animal experimental procedures were strictly conducted in accordance with the established guidelines for animal experiments and had received approval from the Animal Ethics Committee of Shanghai University (ECSHU 2025–196).

## Declaration of competing interest

The authors declare that they have no known competing financial interests or personal relationships that could have appeared to influence the work reported in this paper.

## Data Availability

Data will be made available on request.
