# Supplementary material for: Cartilage-targeting engineered probiotic bacterial extracellular vesicles treat osteoarthritis through modulation of matrix homeostasis
Source: Mater Today Bio. 2026 Sep 24;41:103668. doi: 10.1016/j.mtbio.2026.103668 (PMC13635493; doi:10.1016/j.mtbio.2026.103668)
Supplement: Supplementary file 1 [file mmc1.docx]

**Table 1**. Mouse PCR primer sequences.

| Gene | Forward primer (5’-3’) | Reverse primer (5’-3’) |
| --- | --- | --- |
| *ColⅡ* | CAG GAA TTT GGT GTG GAC ATA GGG | TCA GGT CAG GTC AGC CAT TCA G |
| *Aggrecan* | CTGATCCACTGTCCAAGCACCATG | ATCCACGCCAGGCTCCACTC |
| *SOX9* | TCAACGGCTCCAGCAAGAACAAG | CTCCGCCTCCTCCACGAAGG |
| *MMP13* | CAG TTG ACA GGC TCC GAG AAA TG | CAC ATC AGG CAC TCC ACA TCT TG |
| *ADAMTS5* | AAC AGG AGG ATC ATC GCA GAT ACA G | GAC TCG TGG TGA CAA TGA CAT GAA G |
| *COL1a* | AGGGCCAAGACGAAGACATC | AGGGCCAAGACGAAGACATC |
| *IL 1β* | CTCACAGCAGCATCTCGACAAGAG | TCCACGGGCAAGACATAGGTAGC |
| *TNF-α* | TTAGGAACGTGGATGAGACC | ATGCTGCTGTTCAGCTCTTC |
| *GADPH* | AGA AGG TGG TGA AGC AGG CAT C | CGA AGG TGG AAG AGT GGG AGT TG |

Figure S1. The isolated primary cells were identified as chondrocytes by flow cytometry analysis.

Figure S2. (A) Representative fluorescence images of chondrocytes treated with different inhibitors for 1 h, followed by incubation with PKH26-labeled BEVs for 24 h. Wortmannin inhibits micropinocytosis. Chlorpromazine inhibits clathrin-mediated endocytosis. Genistein inhibits caveolae-mediated endocytosis. Nystatin inhibits lipid raft-dependent uptake. Scale bar = 20 μm. (H) Semi-quantitative analysis of relative fluorescence intensity of BEVs.

Figure S3. (A) Representative 2D micro-CT images depicting subchondral bone microstructure. (B) 3D reconstructed images visualizing osteophytes. (C) Quantification of total osteophyte volume per joint. (D) Representative images of H&E-stained mouse knee joint sections. (E) quantitative scores of synovitis. Scale bar = 500 μm.

Figure S4. (A) Cellular uptake of PKH26-stained W-LGG-EVs. Scale bar =25μm. (B) H&E staining of major organs (heart, liver, spleen, lung, and kidney) following administration of PBS, LGG-EVs, and W-LGG-EVs. Scale bar = 100 μm.

Figure S5. (A) Analysis of fluorescence intensity in major organs (heart, liver, spleen, lung, kidney) and knee joints across experimental mouse groups. (B) Fluorescence images of fibroblasts incubated with PKH26-labeled BEVs and W-BEVs for 24 h. Scale bar = 25 μm. (C) Semi-quantitative analysis of relative fluorescence intensity for BEVs versus W-BEVs. Data are presented as mean ± SD; n = 3. *p < 0.05, **p < 0.01, ***p < 0.001.

Figure S6. Specific inflammatory factors were assessed by RT-qPCR at indicated time points. Data are presented as mean ± SD; n = 3. *p < 0.05, **p < 0.01, ***p < 0.001.

Figure S7. (A) Immunofluorescence of COLⅡ and MMP13 expression in the IL-1β-induced chondrocytes treated with BEV and W-BEV. Scar bar=200 μm.（B）Semi-quantitative analysis of relative fluorescence intensity for BEVs versus W-BEVs. Data are presented as mean ± SD; n = 3. *p < 0.05, **p < 0.01, ***p < 0.001.

Figure S8.(A) Representative images of OA chondrocyte migration capacity at 24 hours post-scratching, as demonstrated by the scratch assay. Scale bar = 500 μm.

(B) Quantification of residual scratch wound area across experimental groups. Data are presented as mean ± SD; n = 3. *p < 0.05, **p < 0.01, ***p < 0.001.

Figure S9. (A) The effect of BEV and W-BEV on cell proliferation by (5-ethynyl-2’-deoxyuridine, EdU) staining. (B) The statistical results of EdU staining. Scale bar = 200 μm. Data are presented as mean ± SD; n = 3. *p < 0.05, **p < 0.01, ***p < 0.001.

Figure S10.(A) Representative hematoxylin and eosin (H&E)-stained images of articular cartilage. Scale bar = 100 μm. （B）Ratio of HC and CC thickness. Data are presented as mean ± SD; n = 3. *p < 0.05, **p < 0.01, ***p < 0.001.
